# Flanking Support: How Subsidiary Cells Contribute to Stomatal Form and Function

**DOI:** 10.3389/fpls.2020.00881

**Published:** 2020-07-02

**Authors:** Antonia Gray, Le Liu, Michelle Facette

**Affiliations:** Department of Biology, University of Massachusetts Amherst, Amherst, MA, United States

**Keywords:** stomata, subsidiary cell, guard cell, plant development, cell division

## Abstract

Few evolutionary adaptations in plants were so critical as the stomatal complex. This structure allows transpiration and efficient gas exchange with the atmosphere. Plants have evolved numerous distinct stomatal architectures to facilitate gas exchange, while balancing water loss and protection from pathogens that can egress via the stomatal pore. Some plants have simple stomata composed of two kidney-shaped guard cells; however, the stomatal apparatus of many plants includes subsidiary cells. Guard cells and subsidiary cells may originate from a single cell lineage, or subsidiary cells may be recruited from cells adjacent to the guard mother cell. The number and morphology of subsidiary cells varies dramatically, and subsidiary cell function is also varied. Subsidiary cells may support guard cell function by offering a mechanical advantage that facilitates guard cell movements, and/or by acting as a reservoir for water and ions. In other cases, subsidiary cells introduce or enhance certain morphologies (such as sunken stomata) that affect gas exchange. Here we review the diversity of stomatal morphology with an emphasis on multi-cellular stomata that include subsidiary cells. We will discuss how subsidiary cells arise and the divisions that produce them; and provide examples of anatomical, mechanical and biochemical consequences of subsidiary cells on stomatal function.

## Introduction: What Is a Subsidiary Cell?

Subsidiary cells are non-guard cells within the stomatal complex. But how do we determine which cells are subsidiary cells? Guard cells flank the stomatal pore and therefore are easily identified. Guard cells have rightly been the focus of scientific inquiry into stomatal function. Turgor-driven movements of guard cell pairs regulate stomatal aperture, and over the last two decades our knowledge of guard cell function has improved dramatically (reviewed in Munemasa et al., 2015; Eisenach and De Angeli, 2017; Jezek and Blatt, 2017). However, relatively little progress has been made toward understanding the role of subsidiary cells. Moreover, identifying and defining exactly which cells comprise the stomatal complex (and even which plants possess them) has proven non-trivial. Taxonomists, anatomists, physiologists, and developmental biologists are likely to have different perspectives on what defines a subsidiary cell. We best identify as developmental biologists, but in this review attempt to synthesize information on subsidiary cells from several perspectives. We choose to define subsidiary cells broadly: cells that are adjacent to guard cells (but not necessarily touching) and are distinct from other epidermal cells. “Distinct” is most easily identified by a unique morphology, but may also be identified by a unique molecular signature. As part of the stomatal complex, subsidiary cells may support guard cell function – but how subsidiary cells do this is likely to be varied and may be biochemical, mechanical or anatomical. In fact, in many cases the definition is taxonomic, but without a complete understanding of the physiological contributions subsidiary cells offer guard cells, a precise definition is difficult.

Ambiguity in subsidiary cell identification is not a recent development. Pant defines a subsidiary cell as any cell that is “recognizably modified” and touching a guard cell; he calls specialized cells surrounding the subsidiary that do not touch a guard cell an “encircling cell” (Pant, 1965). In her classic textbook, Esau identifies subsidiary cells as those that “appear to be associated functionally […] and are morphologically distinct from other epidermal cells” (Esau, 1965) and may include cells that do not touch. Unfortunately, even these relatively simple definitions can be ambiguous or conflicting – both rely on subjective assessments of whether a subsidiary cell has a “distinct” or “recognizably modified” morphology. We use language consistent with Tomlinson (1969) and term all of these subsidiary cells. We consider any cell associated with guard cells that has an identity distinct from neighboring cells a subsidiary cell. A distinct identity can be defined not only by a unique morphology, but by a unique molecular signature (such as genes or proteins expressed). The division sequence of subsidiary cells may produce anatomically distinct cells. In our inclusive definition, we consider taxonomic and anatomical contributions as important as physiological contributions, but realize as our understanding of subsidiary cell function expands more refined definitions will likely develop.

**FIGURE 1:**
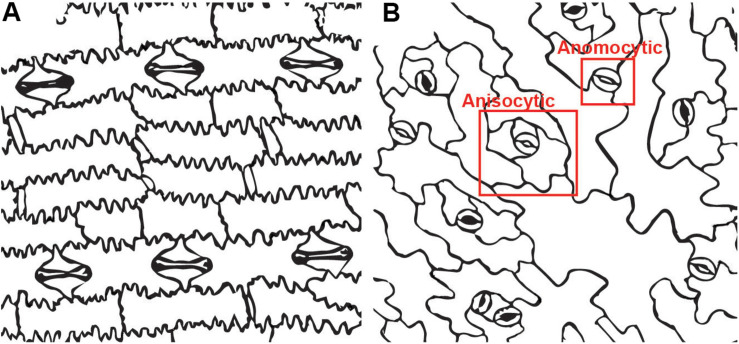
Stomatal complexes in two model systems. **(A)** *Zea mays* (maize) has paracytic stomata. The subsidiary cells dominate the stomatal complex while the guard cells are a pair of small dumbbell shaped cells in the center.**(B)** *Arabidopsis thaliana* has both anisocytic stomata with three subsidiary cells and anomocytic stomata with no subsidiary cells. The anisocytic stomatacan be difficult to detect, since the subsidiary cells are variable in size.

Contrasting the stomatal apparatus between the model systems *Arabidopsis thaliana* and *Zea mays* highlights some of the difficulty in identifying subsidiary cells. In many cases it is simple to identify morphologically distinct cells flanking the guard cells, such as the case in *Z. may*s (corn or maize). In *Z. mays* and other grasses subsidiary cells are always in pairs flanking the guard cells, are uniquely shaped, are more pectin-rich and are therefore readily identified (Figure 1A). However, in the *Brassicaceae* – which includes the model species *A. thaliana* – subsidiary cells are subtly different from epidermal cells. The subsidiary cells are unequal in size and variable in shape, making them difficult to identify (Figure 1B). Not every stomatal complex within the same *A. thaliana* leaf includes subsidiary cells (Nadeau and Sack, 2002). This morphological ambiguity has led to disagreement as to whether *A. thaliana* has subsidiary cells at all (Serna and Fenoll, 2000; Nunes et al., 2020). Given the subtle shape differences in putative subsidiary cells in *A. thaliana*, molecular markers may be a good way to identify subsidiary cells. Gene-specific expression may be considered evidence supporting an identity distinct from other epidermal cells, which may in turn be indicative of a unique function. PATROL1 controls protein trafficking including that of the plasma membrane proton pump AHA1, which is important for guard cell function (Hashimoto-Sugimoto et al., 2013). *PATROL1* is expressed in guard cells and a subset of adjacent cells – which are subsidiary cells (Higaki et al., 2014). Since not all guard-cell adjacent cells express *PATROL1*, but rather it appears in the smaller cells previously identified as subsidiary cells, this indicates these cells have a unique molecular identity and should be considered part of the stomatal complex. Additional molecular markers of subsidiary cell fate will help clarify if (and which) guard-cell adjacent cells have identities distinct from other epidermal cells, but none are currently known in *A. thaliana*. In *Z. mays*, where subsidiary cells are morphologically obvious, there are potential molecular markers of subsidiary cell identity. A SWEET-family protein is expressed in subsidiary cells (Wang et al., 2019b). A gene encoding a specific *Shaker-*family potassium channel is also specifically expressed in maize subsidiary cells (Büchsenschütz et al., 2005). Whether expression of these genes – and subsidiary cell identity in general – is conserved across species is unknown. We predict that while some characteristics might be preserved, there is likely to be a large variation in the molecular components within subsidiary cells since they are varied in morphology, size, and ontogeny. A more thorough understanding of subsidiary cell function will help in accurate classification.

## What Do Subsidiary Cells Look Like?

Subsidiary cells vary widely in number, arrangement and potential function. The diversity in stomatal apparatus morphology is due primarily to diversity in subsidiary cell features, which has led to accepted definitions of subsidiary cell arrangements. Stomatal terminology was originally associated with certain taxonomic groups; thus, the language of stomatal subtypes is elaborate. It can be confusing at best, and conflicting at times. Our coverage of stomatal complexes will not be exhaustive; rather we will highlight stomatal patterns that either illustrate different ontogenies or stomatal morphologies, especially those that we feel are interesting from a developmental perspective or highlight physiological contributions. A recent survey of stomatal complex morphologies, from a variety of monocot plant lineages and their cell divisions, is reviewed in Rudall et al. (2013). Texts that cover stomatal complex morphology that we have found particularly informative include: (Pant, 1965; Tomlinson, 1969, 1974; Fryns-Claessens and Van Cotthem, 1973; Ziegler, 1987; Prabhakar, 2004; Carpenter, 2005).

**FIGURE 2:**
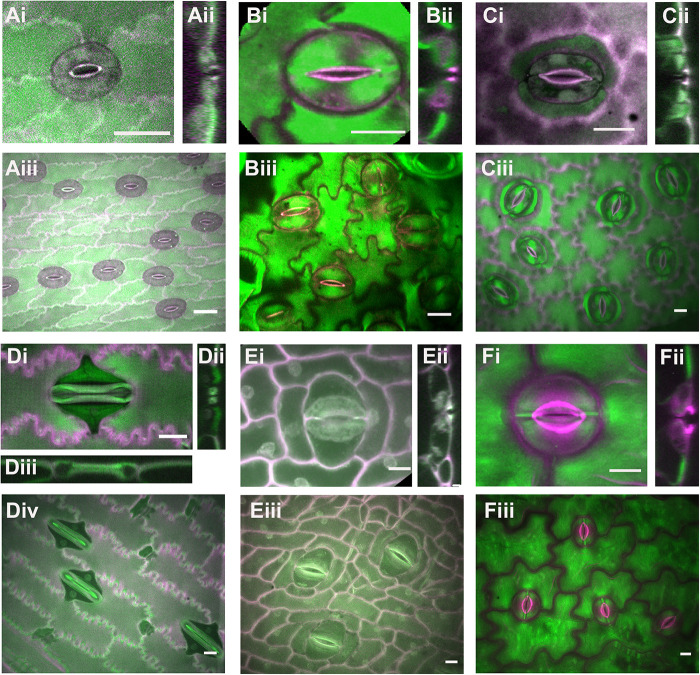
Stomatal complex types, part 1. All images are imaged via confocal microscopy. Images **(i,iii)** are full or partial z-projections while image **(ii)** is a 3D-reconstructed side view through the stomatal pore. **(A)** *Selaginella uncinata* – anomocytic. **(B)** *Solanum* spp. (wild tomato) – anomocytic. **(C)** *Coffea rubiaceae* (coffee) – paracytic **(D)** *Zea mays* (maize/corn) – paracytic **(E)** *Musa acuminate* (banana) – paracytic **(F)** *Dianthus chinensis*; diacytic. Green = Calcofluor White and Magenta = Direct Red, except for **(Di,Dii)** where Green = Aniline Blue and Magenta = Direct Red. All scale bars are 15 micrometers.

**FIGURE 3:**
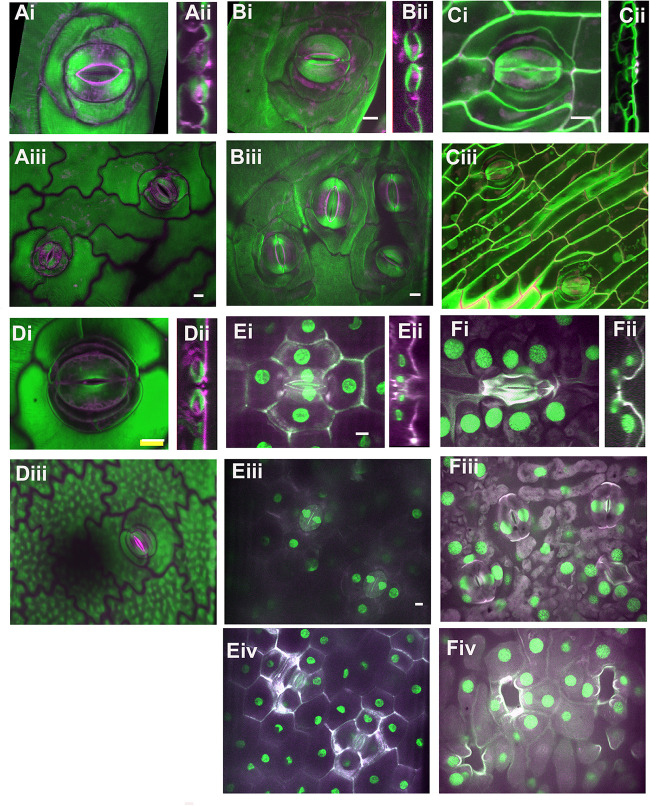
Stomatal complex types, part 2. All images are imaged via confocal microscopy. Images **(i,iii)** are full or partial z-projections while image **(ii)** is a 3D-reconstructed side view through the stomatal pore. For sunken stomata in *Agave* and gingko **(E,F)** a lower focal plane containing the guard cells **(iii)** and a higher focal plane showing epidermal and subsidiary cells **(iv)** are shown. **(A)** *Kalanchoe* spp. (common unknown variety from garden center); anisocytic and heliocytic. **(B)** *Begonia spp*. (common unknown variety from garden center) – heliocytic **(C)** *Didierea madagascariensis* - unusual type **(D)** *Anacampseros rufescens* – tetracytic with four lateral subsidiary cells **(E)** *Agave bracena* – tetracytic with 2 lateral and 2 polar subsidiary cells. **(F)** *Ginkgo biloba* – cyclocytic. Green = Calcofluor White and Magenta = Direct Red, except for **(E,F)** where Green = Propidium Iodide and Magenta = Calcafluor White. All scale bars are 15 micrometers.

**FIGURE 4:**
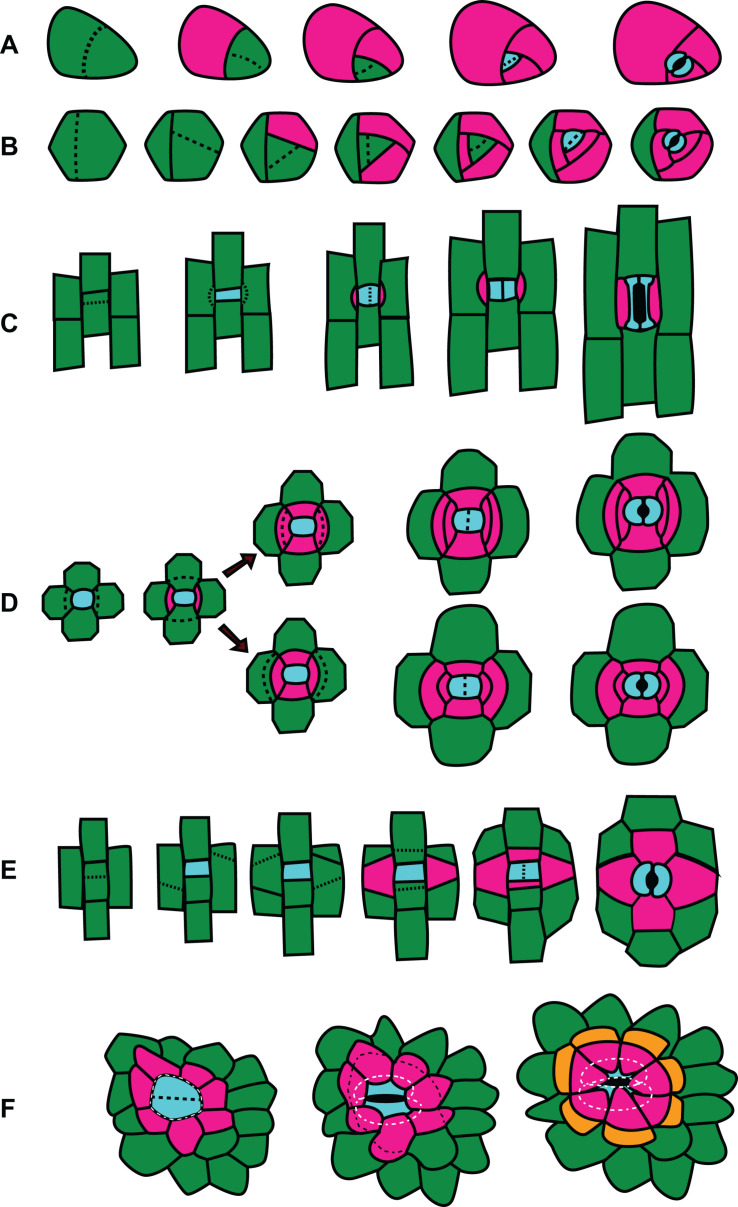
Division patterns in stomata. Divisions outlined left (youngest cells) to right (oldest cells). GMC and guard cells are cyan; subsidiary cells are pink or orange; other cells are green. **(A)** Amplified anisocytic divisions. This type of division is common among eudicots such as *Arabidopsis thaliana*. **(B)** Helicocytic divisions. This type of stomatal complex is seen in some eudicots such as begonia. **(C)** Gramineous (grass) divisions. Paracytic divisions, as seen in other monocots like lily or *Tradescantia virginiana*, follow the same pattern but have kidney shaped guard cells rather than the dumbbell shaped guard cells characteristic of grasses. **(D)** Two possible patterns of hexacytic stomatal generation. The generation of lateral subsidiaries is identical, however in the upper panel the cell closest to the GMC divides, while in the lower panel the cell distal from the GMC divides. **(E)** Tetracytic divisions in *Agave* spp. and some other monocots and feature unusual oblique divisions in the formation of the lateral subsidiary cells. **(F)** Recruitment of subsidiary cells in cyclocytic gingko.

Examples of known stomatal morphologies imaged via confocal microscopy, including reconstructed side views through the stomatal pore, are in Figures 2, 3. Division patterns to achieve different stomatal morphologies are in Figure 4. Lateral subsidiary cells run parallel to the stomatal pore whereas polar subsidiary cells are perpendicular to the stomatal pore. Stomata that have no discernable subsidiary cells are called anomocytic, such as those in *Selaginella uncinata* (Figure 2A). Previously, anomocytic stomata were termed ranunculaceous (Metcalfe and Chalke, 1957). *A. thaliana* has both anomocytic stomata and anisocytic stomatal complexes (Figure 1A). Anisocytic stomatal complexes have three unequally sized subsidiary cells associated with the guard cell pair, where one of these three cells is smaller than the other two. Previously, anisocytic stomata were termed cruciferous because this arrangement is typical of crucifers such as *A. thaliana* (Metcalfe and Chalke, 1957). Wild tomato (*Solanum spp.)* may also have both anomocytic and anisocytic stomata (Figure 2B; Sampaio et al., 2014). Comparison of the physiological responses of different adjacent stomata – those with and without subsidiary cells – in species such as *A. thaliana* or tomato would help illustrate the functional contributions of subsidiary cells in a species where only subtle morphological differences exist.

Stomatal complexes with a pair of lateral subsidiary cells are called paracytic (previously rubiaceous) (Metcalfe and Chalke, 1957). *Coffea rubiacea* (coffee) has paracytic stomata (Figure 2C). Grass stomata are not only paracytic, but also have dumbbell-shaped guard cells and therefore are termed Graminacious (Figure 2D). The contributions of subsidiary cells in grasses are arguably the best studied (e.g., Raschke and Fellows, 1971; Majore et al., 2002; Raissig et al., 2017; Wang et al., 2019b). The subsidiaries align along the outer edge of the guard cell and maintain the symmetrically parallel arrangement of guard cells. It is easy to imagine how the extended cell-cell contact might help support guard cells mechanically and biochemically. Graminaceous stomata have been recognized for their rapid movements, which is thought to be attributable to both their paracytic subsidiary cells and the unique shape of the guard cells (Johnsson et al., 1976; Franks and Farquhar, 2007; Vico et al., 2011). *Musa acuminata* (banana) stomata are an example of how stomatal form can be difficult to classify (Figure 2E). A pair of obvious lateral subsidiary cells indicate paracytic stomata; however, in some cases it appears there may be a pair of polar subsidiary cells as well, or in some cases even up to six subsidiaries (hexacytic). In side view, lateral subsidiary cells overarch the stomatal pore, whereas none of the other adjacent cells do so; hence we predict all these stomatal complexes are paracytic. As-of-yet unidentified molecular markers would help clarify these cells’ identities.

Stomata with a pair of polar subsidiary cells perpendicular to the guard cell pore orientation are called diacytic (previously caryophyllaceous), such as those in *Dianthus chinensis* (Figure 2F). Note the cuticular ledges of *D. chinensis* (magenta) that are set back from the pore (Figure 2Fi) and can be seen in side view (Figure 2Fii). In contrast, these cuticular ledges are quite close to the center of the pore in coffee and tomato.

**FIGURE 5:**
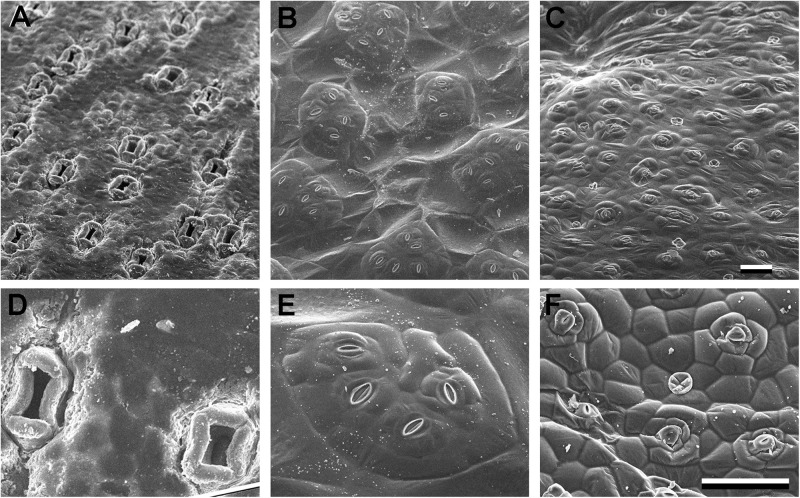
Sunken and raised stomata **(A–D)** *Agave bracena*. **(B–E)** *Begonia* spp. (common house plant), **(C,F)** *Pellionia repens* (Trailing watermelon begonia). Panels **(A–C)** are identical scale; **(D–F)** are identical scale. Scale bars are 100 microns.

More complicated stomatal architectures are shown in Figure 3. Subsidiary cells in *Kalanchoe* spp. are easy to identify and this plant displays several stomatal types within a single leaf (Figure 3A). Figure 3Ai shows an anisocytic stomatal complex, but often stomatal complexes with a spiral pattern of additional subsidiary cells can be observed, such as in the upper right corner of Figure 3Aiii. This spiraling pattern is termed heliocytic (Fryns-Claessens and Van Cotthem, 1973); although spiral stomatal complexes in *Kalanchoe* spp. have been otherwise classified (Inamdar and Patel, 1970; Xu et al., 2018; Nunes et al., 2020). Stomata in *Begonia* spp. are likewise heliocytic (Figure 3B) and may be found in clusters or individually (Figure 5). The pattern of cell divisions that produce anisocytic and heliocytic stomata are initially similar (discussed in the section below) therefore it is notable that *Kalanchoe* spp. has both stomatal architectures. Stomatal complexes of *Didierea madagascariensis* are unique; they may be paracytic but often will have additional C-shaped subsidiary cells (Figure 3C).

Stomata with four stomata are often termed tetracytic, although the cell arrangements vary. For example, *Anacampseros rufescens* has 4 lateral subsidiary cells (Figure 3D) while *Agave bracena* has 2 lateral and 2 polar subsidiaries (Figure 3E). The stomata of *Agave* are dramatically sunken, as seen in the side view in Figure 2Fii. The guard cell pair lies well below the epidermal surface, and the subsidiary cells extend upward to create the walls of the pore, and the cuticular stomatal ledges are on the subsidiary cells. Here, the main contribution of subsidiary cells is perhaps anatomical. Likewise, the gymnosperm *Ginkgo biloba* has sunken stomata (Figure 3F). The many subsidiary cells of gingko are variable in number and are arranged in a circular pattern that reaches over the recessed guard cells, which is called cyclocytic.

Even without a thorough examination of all the possible stomatal complex arrangements, the terminology is dense and classification can become challenging. Within some families stomatal morphology is highly conserved while in others it can be quite variable (Baranova, 1992). This variability, coupled with the difficulty in identifying subsidiary cells, led to the suggestion that the division patterns leading to stomatal complex formation is a more accurate classification system because the division sequence is more conservative than the final structure (Rasmussen, 1981; Ziegler, 1987). Stomatal ontogeny – that is, the divisions that generate stomata – are distinct from stomatal complex classification based on subsidiary cell arrangement.

## How Do Subsidiary Cells Arise?

Plant stomatal complexes are derived from a carefully controlled series of asymmetric cell divisions (Sack, 1987; Facette and Smith, 2012; Torii, 2015; Shao and Dong, 2016; Simmons and Bergmann, 2016; Chater et al., 2017). Stebbins and Shah noted the importance of understanding the mechanisms behind stomatal complex formation, and the utility of studying them as a model system for asymmetric cell divisions 60 years ago (Stebbins and Shah, 1960). Stomatal divisions have been used as a model for asymmetric division in large number of model systems as they present opportunities to study cell polarity, cell-cell communication, and cell division (Pickett-Heaps, 1969; Zeiger and Stebbins, 1972; Palevitz and Hepler, 1974; Apostolakos and Galatis, 1987; Cleary, 1995; Geisler et al., 2000; Shpak et al., 2004; MacAlister et al., 2007; Dong et al., 2009; Hunt and Gray, 2009; Chater et al., 2016; Raissig et al., 2016).

Generation of guard cell pairs occurs in a stereotypical fashion. A protodermal cells in the epidermis of immature leaves differentiates into a meristemoid mother cells (MMC); the MMC divides asymmetrically to give a small meristemoid and a stomatal lineage cell. The meristemoid differentiates into a guard mother cell (GMC), which divides via a symmetric oriented division to yield the two guard cells. Prior to the division of the GMC, subsidiary cells (if present) arise. Subsidiary cells may be generated via divisions of the meristemoid or MMC, in which case they are termed mesogenous (Metcalfe and Chalke, 1957). In mesogenous stomata the subsidiary cells and guard cells are derived from the same cell lineage. In other cases, protodermal cells adjacent to the meristemoid or GMC are recruited, and subsidiary cells therefore are derived from a lineage that is distinct from the guard cells. In this case, subsidiary cells are of perigenous origin (Metcalfe and Chalke, 1957). Necessarily, stomata of perigenous origin require cell-cell communication with the neighboring epidermal cells – very often particular neighbors on certain sides of the GMC – that are recruited into the stomatal complex. Mesoperigenous stomata have both subsidiary cells that arise from the same stomatal lineage as the GMC and subsidiary cells that are recruited from neighboring cells. More detailed and complex subclassifications of stomatal ontogenies exist (Pant, 1965; Tomlinson, 1974; Rasmussen, 1981). Regardless of whether the stomatal complex is of mesogenous or perigenous origin, the number of times a cell divides (in addition to which cells divide) has ramifications for final stomatal morphology.

A few contrasting examples of stomatal divisions are provided in Figure 4. Many reviews regarding *A. thaliana* cell divisions as well as the molecular factors (transcriptional regulators, signaling and scaffolding molecules, and cell cycle regulators) exist and thus will not be covered extensively here. Many fate factors appear to be conserved across phyla (Harris et al., 2020). The divisions that create anisocytic stomata such as those in *A. thaliana* are illustrated in Figure 4A. An asymmetric division of a meristemoid mother cell produces a small meristemoid and a larger stomatal lineage cell. Anisocytic stomata with three subsidiary cells are created when an “amplifying division” occurs (Pillitteri and Dong, 2013). The meristemoid divides asymmetrically two more times, creating surrounding subsidiary cells (Figure 4A). The GMC finally divides symmetrically to form a pair of guard cells surrounded by three subsidiary cells. Anomocytic stomata are formed in *A. thaliana* when amplifying divisions are absent. Expression patterns of the PATROL1 in mature leaves (Higaki et al., 2014) suggest perhaps the stomatal lineage cell that is sister to the meristemoid may acquire subsidiary cell identity, although a careful analysis of cell lineage and PATROL1 expression in the same leaf needs to be performed to confirm this.

In certain stomata the meristemoid undergoes additional divisions to form a concentric ring of subsidiary cells to form a heliocytic stomatal complex (Figure 4B; Rudall et al., 2018). Heliocytic stomata are observed in some species of begonia and follow a very similar developmental pattern to those in *A. thaliana*, but with additional regenerations of the meristemoid. Interestingly, the meristemoid is often the larger of the two daughter cells after a division in this type of stomatal complex, which is highly unusual (Rudall et al., 2018). The pattern created by the amplified divisions form a spiral that raises the stoma above the leaf surface (Figure 4B). Contrasting the divisions of anomocytic stomata in Figure 4A and heliocytic stomata in Figure 4B, highlights the additional rounds of successive divisions of the meristemoid prior to it’s differentiation to a GMC. In *A. thaliana* the transcription factor MUTE controls the transition from meristemoid to GMC (Pillitteri et al., 2007). In *A. thaliana mute* mutants, excessive rounds of asymmetric meristemoid divisions produce a cluster of cells that look similar to mid-developmental stages of heliocytic stomata depicted in Figure 4B – however, *mute* mutants arrest at this stage. In heliocytic stomata the meristemoid successfully differentiates into a GMC, which then goes on to undergo a successful single oriented division. Comparing the expression and function of MUTE in heliocytic stomata o*f Begonia* or in *Kalanchoe*, which possesses both heliocytic and anisocytic stomata, is likely to provide insights into the developmental mechanisms of different cell patterns.

Stomatal divisions in the grasses *Z. mays, Oryza sativa*, and *Brachypodium distachyon* and the monocot *Tradescantia virginiana* have been used as models and undergo an identical division sequence (Figure 4C; Cleary, 1995; Facette and Smith, 2012; Apostolakos et al., 2018; Hepworth et al., 2018; Nunes et al., 2020). A meristemoid mother cell within a stomatal cell file undergoes an asymmetric division to produce a guard mother cell and a sister interstomatal cell. Stomatal divisions in grasses are perigenous; the subsidiary mother cells (SMCs) are recruited from adjacent protodermal cells. Presumably, there is an inductive signal sent from the GMC to lateral neighboring protodermal cells that stimulates them to become SMCs. The SMCs polarize toward the GMC and each SMC divides asymmetrically – exactly once – to give a small subsidiary cell and larger pavement cell. It is presumed that the GMC sends out a polarizing cue that induces the adjacent protodermal cells to differentiate, polarize and divide asymmetrically (Stebbins and Shah, 1960). Once the subsidiary cell is formed, the GMC undergoes its final symmetric division. In both *A. thaliana* and grasses, fate regulators SPEECHLESS, ICE/SCRM, MUTE, and FAMA are important for stomatal development, but play subtly different roles (Liu et al., 2009; Raissig et al., 2016, 2017; Wang et al., 2019a). The transcription factor MUTE is important in *A. thaliana* for specifying GMC identity but in *B. distachyon* and *Z. mays* is important for subsidiary cell differentiation as well. BdMUTE is produced in the GMC and moves, presumably through plasmodesmata, to adjacent subsidiary mother cells (Raissig et al., 2017; Wang et al., 2019a). MUTE might be the polarizing cue that induces adjacent protodermal cells to differentiate into SMCs, divide and polarize. Polarity markers accumulate in or at the plasma membrane of the SMC adjacent to the GMC, with the branched-actin regulator BRK1 polarizing immediately after GMC formation (Facette et al., 2015). Is MUTE the inductive signal the GMC sends to the neighboring cell, that induces expression or localization of these polarity factors? Since BRK appears polarized so soon after the meristemoid-generating division, this means MUTE must travel even earlier. Determining the relative timing of MUTE-BRK appearance/polarization in SMCs, and whether one is dependent on the other, will help crystallize our understanding of the process of SMC recruitment in grasses.

Consider the potential role of factors known to be important in grass divisions in the formation of certain tetracytic stomata – those that have two lateral and two polar subsidiary cells. Often, the lateral subsidiary cells form via an asymmetric division of recruited neighboring cells similar to that seen in grasses – perhaps MUTE also shows cell-to-cell movement in these tetracytic stomata. Cell-to-cell movement of MUTE does not occur in *A. thaliana*, which does not recruit neighboring cells; it would be interesting to know if the same movement occurs in other plants that have perigenous divisions or if other mechanisms evolved. Likewise, investigation of whether SMC-polarized proteins important for subsidiary-generating divisions in grasses such as BRK1 (Facette et al., 2015) or receptor-like proteins PAN1 and PAN2 (Cartwright et al., 2009; Zhang et al., 2012) also polarize in lateral SMC recruitment would indicate if common or independent mechanisms stimulate perigenous divisions in different plants. These tetracytic stomata also have polar subsidiary cells that are generated via an asymmetric division of stomatal lineage cells lying in the opposite orientation of the lateral subsidiary cells (Tomlinson, 1974). Therefore, these tetracytic stomata form via 2 additional divisions that grasses do not undergo but are otherwise similar. Do the same factors play a role in the mesogenous division? For example, in tetracytic stomata, is MUTE traveling to polarly adjacent cells in addition to laterally adjacent cells? If so, why does MUTE only travel to the lateral protodermal cells (and not the polar cells) in grasses to induce SMC fate? Notably, ectopic overexpression of BdMUTE in *B. distachyon* results in many excess divisions throughout the epidermis; but up to 4 layers of cells surrounding the guard cells appear as if they may be associated subsidiary cells in both lateral and polar directions (Raissig et al., 2017). This suggests that if BdMUTE is present in the polar cells, it is sufficient for subsidiary cell fate, and its movement or stability is somehow regulated. Markers of terminal subsidiary cell fate in *B. distachyon*, coupled with examination of MUTE localization in species with tetracytic stomata could shed light on how the diversity of stomatal form is achieved.

In the tetracytic stomata of *A. rufescens* (Figure 3D) there are no polar subsidiary cells and instead there are two pairs of lateral subsidiary cells. It is easy to imagine how the pair of subsidiary cells closest to the guard cells could be generated in a manner like grasses, where adjacent protodermal cells are recruited and divide asymmetrically. But how do the outer pair of subsidiary cells arise? The initial division of a SMC adjacent to the SMC would give a small subsidiary cell and a larger pavement cell – but then which of these cells divides to give another subsidiary cell? We don’t know the answer in the case of *A. rufescens*, but Tomlinson (1974) showed that in hexacytic stomata, either scenario is possible. Hexacytic stomata found in the *Geogenanthus* and *Commelina* have two pairs of lateral subsidiary cells, as well as a pair of polar subsidiary cells (Figure 4D). In *Geogenanthus*, after an initial asymmetric division of the lateral SMC, the smaller cell divides again. Reciprocally, in *Commelina*, the larger daughter divides again. The patterns of division are conserved within families, indicating different evolutionary paths.

During all stomatal divisions described thus far, one division is required to form one subsidiary cell. *Agave* spp. initiates a meristemoid in a manner similar to other monocots, but then lateral subsidiary cells are formed via two unusual oblique asymmetric divisions that result in trapezoid shaped lateral subsidiary cells (Tomlinson, 1974). Therefore two coordinated divisions are required to make a single subsidiary cell – a developmental process that seems fundamentally different from a single division. Since the lateral subsidiary cell are recruited (as in grasses) from an adjacent row of non-stomatal lineage cells, they are of perigenous origin. On the other hand, polar subsidiary cells are generated from an asymmetric division of stomatal lineage cells (Figure 4F). This is an example of a mesoperigenous stomatal complex.

There are some unusual cases of stomata with many subsidiary cells arranged radially but without the spiral amplifying division pattern seen in begonia (Carpenter et al., 2005). *Banksia conferta* has these rare actinocytic stomata where subsidiary cells are recruited from neighboring protodermal cells. Here, a presumptive cue from the GMC induces differentiation but no cell division. These stomatal complexes develop in such a way that the subsidiary cells underlie the guard cells to a degree, pushing the stoma above the leaf epidermis. *Platanus orientalis* has a similar stomatal complex and appears to produce stomatal clusters (Carpenter et al., 2005). It has been suggested that actinocytic stomata are simply a variant of anomocytic (no subsidiary cells) stomata (Stace, 1965). Because these cells raise the guard cells within the epidermis, they have a unique anatomical contribution to the stomata. The recruited cells are likely to have a unique molecular signature since they differentiate differently than other epidermal cells and we therefore consider them subsidiary cells.

Cyclocytic stomata can be observed in both gingko (Figures 3F, 4F) and cycads (Pant and Mehra, 1964). These stomata are very similar to the actinocytic type but the subsidiary cells are above the guard cells rather than below and thus create a sunken stomatal complex. The subsidiary cells also divide leaving smaller, polygonal cells distal to the guard cells (Figure 4C).

## What Do Subsidiary Cells Do?

Form follows function. The diversity in subsidiary cell arrangement and shapes may reflect diverse subsidiary cell function, as well as diverse ways to achieve the same function. Ultimately, the function of the stomatal apparatus is to facilitate gas exchange with the environment. Because plants’ environments vary, stomatal adaptations also vary. We will discuss three potential roles for subsidiary cells: anatomical roles that raise or lower guard cells relative to the epidermal surface, mechanical roles during stomatal movements, and molecular roles involving ion and water flux in the stomatal complex.

Stomata are often not flush with the epidermal surface but rather may lie below or above it. Stomatal crypts are large invaginations in the epidermis spanning many cells, typically containing many stomata and often will also have trichomes. Subsidiary cells do not contribute directly to the formation of crypts, but stomatal crypts and sunken stomata (which rely on subsidiary cell architecture) have several conceptual parallels. It was generally accepted that crypts are an adaptation to limit water loss by increasing the boundary layer and were primarily associated with plants growing in water-limiting conditions (i.e., xerophytes) (Katherine, 1977). However, it has become evident that crypts are more widespread and might not limit water loss (Roth-Nebelsick et al., 2009); although certain morphological features of the crypts may affect whether the crypts are indeed a xeromorphic trait (Jordan et al., 2008). An alternative function of crypts may be that they facilitate diffusion of carbon dioxide in thick leaves (Hassiotou et al., 2009).

Sunken stomata are distinct from stomatal crypts; rather than an invagination or depressed area of the epidermis, just the stomata (or guard cells within the stomatal complex) are below the epidermal surface. Sunken stomata in *Agave* are seen in Figure 3E by confocal microscopy and in Figures 5A,D by scanning electron microscopy. Figure 3Eiii shows a reconstructed side view of the stomata, where the guard cells are well below the rest of the leaf epidermal cells. The subsidiary cells partially cover the pore and extend up above the rest of the epidermal cells. In *Agave* the polar and lateral subsidiary cells are essential to creating the sunken stomatal morphology. Subsidiary cells in gingko (Figure 3F) likewise are essential to creating the recessed stoma. Like stomatal crypts, sunken stomata were thought to be associated with arid climates, but can also be found in humid climates. Sunken stomata are particularly prevalent within the gymnosperms (Sack, 1987) where they can become plugged with wax or cutin. Like crypts, sunken stomata are thought to increase the transfer resistance by increasing the boundary layer; the net effect is less water loss. However, this fails to explain why sunken stomata would be found in humid environments. In a tropical gymnosperm, leaves with plugged stomata actually had a higher stomatal conductance at high vapor pressure deficit than leaves without plugged stomata (Feild et al., 1998). Moreover, plugged stomata had higher maximal photosynthetic rates. This led to the hypothesis that hydrophobic plugs prevent stomata from filling with water in very humid environments (Feild et al., 1998). It is plausible that sunken stomata represent multiple adaptations – although in every case subsidiary cells are integral to obtaining the sunken morphology.

The opposite of sunken stomata are raised or elevated stomata. In *Begonia*, the heliocytic stomata are raised – either in clusters or singly (Figure 5; Papanatsiou et al., 2017; Rudall et al., 2018). The functional significance of raised stomata is unclear, but perhaps it is the reciprocal of sunken stomata – in water-replete conditions it decreases the size of the boundary layer, increasing transpiration. It has been suggested that the raised, clustered stomata in begonia increase the size of the substomatal chamber, facilitating gas exchange within the leaf (Papanatsiou et al., 2017). In begonia, the many subsidiary cells generated by multiple successive rounds of division result in the subsidiary cells creating a base that raises the clustered guard cells up. A different adaptation of raised stomata can be seen in floating leaves of aquatic plants (Ziegler, 1987). The guard cells are supported high on the subsidiary cells, above the epidermal surface, presumably to prevent flooding of the stomatal chamber.

In addition to altering the boundary layer, the morphological arrangement of subsidiary cells in angiosperms affects the mechanical properties of stomata. Turgor-driven guard cell movements are dependent on the wall properties of guard cells. All guard cell walls are thick relative to other epidermal cells, although there is a wall anisotropy that drives stomatal movements. The outer wall is more flexible while the inner wall is thickened and less flexible. In angiosperms in particular, the outer wall distends laterally into neighboring cells during opening. The subsidiary cells are compressed and either displaced laterally and/or basally into the substomatal cavity (Ziegler, 1987). Via mathematical modeling, DeMichele and Sharpe proposed that surrounding epidermal (including subsidiary) cells have a “mechanical advantage” over guard cells (DeMichele and Sharpe, 1973) which was later demonstrated experimentally (Edwards et al., 1976). The mechanical advantage of subsidiary cells is one where turgor pressure of subsidiary cells counterbalances that of guard cells, and subsidiary cell turgor has a greater effect on stomatal aperture than guard cell turgor due to physical properties of the guard cell (DeMichele and Sharpe, 1973; Edwards et al., 1976). Hence, neighboring epidermal cells constrain lateral guard cell movements and limit stomatal opening. Guard cells in non-angiosperms (such as gymnosperms and lycophytes) do not extend laterally into neighboring cells, but rather swell up or down and therefore do not have to overcome the mechanical advantages of neighboring cells (Ziegler, 1987). Using pressure-probe measurements, cryo-SEM imaging and modeling techniques, Franks and Farquhar (Franks and Farquhar, 2007) demonstrated that two species with laterally moving, paracytic guard cells must overcome large mechanical advantages to fully open their stomata. The subsidiary cells were observed to undergo large deformations, and therefore allowing the guard cells to overcome the mechanical advantage of the neighboring cells. One way to achieve these deformations is by altering the osmotic potential of the cells via active transport. Franks and Farquhar suggest a see-sawing mechanism where water and potassium are exchanged, which is discussed more fully below.

An important consideration in the mechanical properties of stomatal complex function is cell wall properties. Subsidiary cells can have different cell wall compositions from other epidermal cells. This is clearly evidenced in maize, where the polychromatic dye Toluidine Blue O stains guard cells blue (which correlates with more lignified walls) but subsidiary cells pink (which correlates with more pectinaceous walls); this has been used as a marker for subsidiary cell fate (Gallagher and Smith, 2000). Based on this staining, the pectinaceous subsidiary cell walls are perhaps more flexible. Recent investigations on the mechanical properties of cell walls have led to insight into guard cell properties and movements (Carter et al., 2017; Yi et al., 2018). Notably, patterns of cellulose in guard cells change during opening and closing (Rui and Anderson, 2016), and cellulose orientation patterns in subsidiary cells appear to run perpendicular to those in guard cells (Shtein et al., 2017). Extending these analyses to both guard and subsidiary cells during stomatal movements would further our understanding of how subsidiary cells support stomatal function. Indeed, Sharpe et al. (1987) point out how differing elastic forces in guard cell and adjacent cell walls are instrumental for stomatal function.

As summarized above, overcoming the mechanical advantage of neighboring cells is likely due to changes in osmotic potential in subsidiary cells. Early studies investigating the mechanism of turgor changes in maize guard cells examined cellular potassium levels cellular by cobaltinitrite precipitation. In open stomata of maize, cellular potassium is high in guard cells while in closed stomata potassium is high in subsidiary cells (Raschke and Fellows, 1971). A reciprocal exchange of potassium between guard and subsidiary cells allows stomatal complexes to overcome the mechanical advantage of neighboring cells, and is also a potential reservoir of water and ions for guard cells. A similar exchange of potassium between guard cells and subsidiary cells has been seen in many other species (Willmer and Pallas, 1972; Dayanandan and Kaufman, 1975). In certain species, potassium is concentrated only in subsidiary cells – and not other epidermal cells touching the guard cell – of closed stomata. However, in other species such as *Selaginella spp.*, which do not have clear subsidiary cells, potassium was seen in many surrounding epidermal cells, up to several cell layers deep. How do we distinguish between an indiscriminate uptake of extracellular potassium pumped out from the guard cell versus an explicit role for subsidiaries in actively exchanging solutes with guard cells? Cell specificity of uptake is one indicator. Raschke and Fellows also examined kinetics to ensure the time scale of subsidiary cell potassium uptake matched stomatal kinetics. However, additional evidence from maize supports subsidiary cell-specific adaptation. Patch clamping (Majore et al., 2002) and gene expression studies (Büchsenschütz et al., 2005) indicate that maize subsidiary cells possess specific potassium channels. A more thorough indexing of any pumps and channels specific to subsidiary cells would strengthen the argument that subsidiary cells indeed undergo an exchange of molecules with guard cells.

The change in potassium levels likely helps drive the turgor changes observed in grass subsidiary cells, but raises several questions. For example, a principle of guard cell identity is that they lose plasmodesmata as part of their development, becoming symplastically isolated. Subsidiary cells, however, maintain their plasmodesmal connections to adjacent epidermal cells (Majore et al., 2002). Under water-limiting conditions, when stomates must be closed, the subsidiary cells must be kept turgid and not lose water and solutes to adjacent epidermal cell. Under water-limiting conditions, failure to keep subsidiary cells turgid would have disastrous consequences, as modeled by Franks and Farquhar (2007). This implicates active mechanisms to maintain subsidiary cell turgor. At least one potassium channel is unique to maize subsidiary cells (Büchsenschütz et al., 2005) but are there other unique channels and pumps? What about other molecules important for guard cell function? Chloride was also seen to shuttle between guard and subsidiary cells (Raschke and Fellows, 1971; Dayanandan and Kaufman, 1975). CST1 is a maize subsidiary cell-specific glucose transporter in the SWEET family that promotes stomatal opening (Wang et al., 2019b). The precise role of CST1 in stomatal regulation is difficult to test but the authors offer several plausible roles for CST1. Proposals include: sequestering glucose in subsidiary cells so it does not induce guard cell hexokinase-induced stomatal closing; increasing the osmolarity of the apoplast via glucose export to decrease subsidiary cell turgor; or providing subsidiary cells with sugar to power their own ion channels. Notably, this gene is duplicated in grasses and the single ortholog in *A. thaliana* to play a role in stomatal function, suggesting a possible grass-specific role.

At least in maize, there are unique transporters within its easily-identifiable subsidiary cells. However, in maize at least some potassium channels are shared by both guard cells and subsidiary cells (Büchsenschütz et al., 2005). Given the observed see-saw localization of potassium, are the same proteins functionally oppositely in guard cells and subsidiary cells, through differential regulation or simply by the existing concentration gradients? In *A. thaliana, PATROL1* is expressed in both guard cells and subsidiary cells. The role of PATROL1 in guard cells includes trafficking the proton pump AHA1 – is PATROL1 AHA1 differentially in these two cell types during opening and closing? Or is PATROL1 trafficking different proteins? Identification of cell-specific and common transporters and regulatory proteins between subsidiary cells versus guard cells should help indicate functional roles and potential regulation of subsidiary cells.

## Conclusion and Outlook

Turgor-driven guard cell movements, and the contribution of subsidiary cells, has been long studied; Heath (1938) identified contributions of cells via puncture experiments nearly 90 years ago. The advent of molecular genetics rapidly exploded our knowledge of guard cell biology, but subsidiary cell biology was ignored. This is likely, at least partially, due to the fact that most experimental advances were accomplished in *A. thaliana*, where subsidiary cells are difficult to identify and do not appear to contribute to the same extent in organisms such as grasses. The rapid stomatal movements of grass stomata are partially attributable to their subsidiary cells, but also due to their unique dumbbell shape considering other species (such as *T. virginiana*) also possess paracytic stomata but are not as rapid (Franks and Farquhar, 2007). Current active research in stomatal development and function in model systems like *B. distachyon, Z. mays*, and *O. sativa* will contribute to understanding of subsidiary cell mechanisms in the economically important grasses (Chen et al., 2016; Hepworth et al., 2018; Nunes et al., 2020). Studies in other models with diverse stomatal architectures like *Begonia* (Rudall et al., 2018) and *Kalanchoe* (Xu et al., 2018) will be just as important. Clearly, the same basic arrangements can be obtained several different ways (e.g., hexacytic stomatal morphology) and similar architectures may have different functions (e.g., sunken stomata). Examination of stomatal complexes in totality, including subsidiary cells, in a diverse array of species will provide a more complete picture of stomatal function. Fortunately, genomic and genetic tools are being developed for a broader array of species meaning we are poised to consider the diversity of stomata examined by botanists and taxonomists.

## Author Contributions

MF and AG wrote the manuscript. MF prepared the Figures 1, 5. LL prepared the Figures 2, 3. AG prepared the Figure 4. All authors contributed to the article and approved the submitted version.

## Conflict of Interest

The authors declare that the research was conducted in the absence of any commercial or financial relationships that could be construed as a potential conflict of interest.

## References

[B1] Apostolakos P. Galatis B. (1987). Induction, polarity and spatial control of cytokinesis in some abnormal subsidiary cell mother cells of Zea Mays. *Protoplasma* 140 26–42. 10.1007/BF01273253

[B2] Apostolakos P. Livanos P. Giannoutsou E. Panteris E. Galatis B. (2018). The intracellular and intercellular cross-talk during subsidiary cell formation in zea mays: existing and novel components orchestrating cell polarization and asymmetric division. *Ann. Bot.* 122 679–696. 10.1093/aob/mcx193 29346521 PMC6215039

[B3] Baranova M. (1992). Principles of comparative stomatographic studies of flowering plants. *The Botanical Review* 58 49–99. 10.1007/BF02858543

[B4] Büchsenschütz K. Marten I. Becker D. Philippar K. Ache P. Hedrich R. (2005). Differential Expression of K+ channels between guard cells and subsidiary cells within the maize stomatal complex. *Planta* 222 968–976. 10.1007/s00425-005-0038-6 16021501

[B5] Carpenter K. J. (2005). Stomatal architecture and evolution in basal angiosperms. *Am. J. Bot.* 92 1595–1615. 10.3732/ajb.92.10.1595 21646077

[B6] Carpenter R. J. Hill R. S. Jordan G. J. (2005). Leaf cuticular morphology links platanaceae and proteaceae. *Int. J. Plant Sci.* 166 843–855.

[B7] Carter R. Woolfenden H. Baillie A. Amsbury S. Carroll S. Healicon E. (2017). Stomatal opening involves polar, not radial, stiffening of guard cells. *Curr. Biol.* 27 2974.e–2983.e. 10.1016/j.cub.2017.08.006 28943087 PMC5640513

[B8] Cartwright H. N. Humphries J. A. Smith L. G. (2009). A receptor-like protein that promotes polarization of an asymmetric cell division in maize. *Science* 323 649–651. 19179535 10.1126/science.1161686

[B9] Chater C. C. Caine R. S. Tomek M. Wallace S. Kamisugi Y. Cuming Y. C. (2016). Origin and function of stomata in the moss physcomitrella patens. *Nat. Plants* 2:16179. 10.1038/nplants.2016.179 27892923 PMC5131878

[B10] Chater C. C. C. Caine R. S. Fleming A. J. Gray J. E. (2017). Origins and evolution of stomatal development. *Plant Physiol.* 174 624–638. 10.1104/pp.17.00183 28356502 PMC5462063

[B11] Chen Z.-H. Chen G. Dai F. Wang Y. Hills A. Ruan Y.-L. (2016). Molecular evolution of grass stomata. *Trends Plant Sci.* 22 124–139. 10.1016/j.tplants.2016.09.005 27776931

[B12] Cleary A. L. (1995). F-actin redistributions at the division site in livingtradescantia stomatal complexes as revealed by microinjection of rhodamine-phalloidin. *Protoplasma* 185 152–165.

[B13] Dayanandan P. Kaufman P. B. (1975). Stomatal movements associated with potassium fluxes. *Am. J. Bot.* 62 221–231.

[B14] DeMichele D. W. Sharpe P. J. H. (1973). An analysis of the mechanics of guard cell motion. *J. Theor. Biol.* 41 77–96. 10.1016/0022-5193(73)90190-2 4754908

[B15] Dong J. MacAlister C. A. Bergmann D. C. (2009). BASL controls asymmetric cell division in Arabidopsis. *Cell* 137 1320–1330. 19523675 10.1016/j.cell.2009.04.018 PMC4105981

[B16] Edwards M. Meidner H. Sheriff D. W. (1976). Direct measurements of turgor pressure potentials of guard cells: ii. the mechanical advantage of subsidiary cells, the spannunqsphase, and the optimum leaf water deficit. *J. Exp. Bot.* 27 163–171. 10.1093/jxb/27.1.163 12432039

[B17] Eisenach C. De Angeli A. (2017). Ion transport at the vacuole during stomatal movements. *Plant Physiol.* 174 520–530. 10.1104/pp.17.00130 28381500 PMC5462060

[B18] Esau K. (1965). *Plant Anatomy.* New York, NY: John Wiley and Sons.

[B19] Facette M. R. Park Y. Sutimantanapi D. Luo A. Cartwright H. N. Yang B. (2015). The SCAR/WAVE complex polarises PAN receptors and promotes division asymmetry in maize. *Nat. Plants* 1:14024. 10.1038/nplants.2014.24 27246760

[B20] Facette M. R. Smith L. G. (2012). Division polarity in developing stomata. *Curr. Opin. Plant Biol.* 15 585–592. 10.1016/j.pbi.2012.09.013 23044038

[B21] Feild T. S. Zwieniecki M. A. Donoghue M. J. Holbrook N. M. (1998). Stomatal Plugs of Drimys Winteri (Winteraceae) protect leaves from mist but not drought. *Proc. Natl. Acad. Sci. U.S.A.* 95 14256–14259. 10.1073/pnas.95.24.14256 9826687 PMC24360

[B22] Franks P. J. Farquhar G. D. (2007). The mechanical diversity of stomata and its significance in gas-exchange control. *Plant Physiol.* 143 78–87. 10.1104/pp.106.089367 17114276 PMC1761988

[B23] Fryns-Claessens E. Van Cotthem W. (1973). A New Classification of the Ontogenetic Types of Stomata. *Bot. Rev.* 39 71–138.

[B24] Gallagher K. Smith L. G. (2000). Roles for polarity and nuclear determinants in specifying daughter cell fates after an asymmetric cell division in the maize leaf. *Curr. Biol.* 10 1229–1232. 11050395 10.1016/s0960-9822(00)00730-2

[B25] Geisler M. Nadeau J. Sack F. D. (2000). Oriented asymmetric divisions that generate the stomatal spacing pattern in arabidopsis are disrupted by the too many mouths mutation. *Plant Cell Online* 12 2075–2086. 10.1105/tpc.12.11.2075 11090210 PMC150159

[B26] Harris B. J. Harrison C. J. Hetherington A. M. Williams T. A. (2020). Phylogenomic evidence for the monophyly of bryophytes and the reductive evolution of Stomata. *Curr. Biol.* 30:2001-2012.e2. 10.1016/j.cub.2020.03.048 32302587

[B27] Hashimoto-Sugimoto M. Higaki T. Yaeno T. Nagami A. Irie M. Fujimi M. (2013). A Munc13-like Protein in Arabidopsis Mediates H + -ATPase translocation that is essential for stomatal responses. *Nat. Commun.* 4 1–9. 10.1038/ncomms3215 23896897 PMC3731666

[B28] Hassiotou F. Evans J. E. Ludwig M. Veneklaas E. J. (2009). Stomatal Crypts May Facilitate Diffusion of CO2 to Adaxial Mesophyll Cells in Thick Sclerophylls. *Plant Cell Environ.* 32 1596–1611. 10.1111/j.1365-3040.2009.02024.x 19627563

[B29] Heath O. V. S. (1938). An experimental investigation of the mechanism of stomatal movement, with some preliminary observations upon the response of the guard cells to ‘Shock.’. *New Phytol.* 37 385–395.

[B30] Hepworth C. Caine R. S. Harrison E. L. Sloan J. Gray J. E. (2018). Stomatal development: focusing on the grasses. *Curr. Opin. Plant Biol. Growth Dev.* 41 1–7. 10.1016/j.pbi.2017.07.009 28826033

[B31] Higaki T. Hashimoto-Sugimoto M. Akita K. Iba K. Hasezawa S. (2014). Dynamics and environmental responses of PATROL1 in Arabidopsis subsidiary cells. *Plant Cell Physiol.* 55 773–780. 10.1093/pcp/pct151 24163289

[B32] Hunt L. Gray J. E. (2009). The Signaling Peptide EPF2 controls asymmetric cell divisions during stomatal development. *Curr. Biol.* 19 864–869. 10.1016/j.cub.2009.03.069 19398336

[B33] Inamdar J. A. Patel R. C. (1970). Structure and development of stornata in vegetative and floral organs of three species of kalanchoe. *Ann. Bot.* 34 965–974.

[B34] Jezek M. Blatt M. R. (2017). The membrane transport system of the guard cell and its integration for stomatal dynamics. *Plant Physiol.* 174 487–519. 10.1104/pp.16.01949 28408539 PMC5462021

[B35] Johnsson M. Issaias S. Brogardh T. And Johnsson A. (1976). Rapid, blue-light-induced transpiration response restricted to plants with grass-like stomata. *Physiol. Plant.* 36 229–232. 10.1111/j.1399-3054.1976.tb04418.x

[B36] Jordan G. J. Weston P. H. Carpenter R. J. Dillon R. A. Brodribb T. J. (2008). The Evolutionary Relations of Sunken, Covered, and Encrypted Stomata to Dry Habitats in Proteaceae. *Am. J. Bot.* 95 521–530. 10.3732/ajb.2007333 21632378

[B37] Katherine E. (1977). *Anatomy of Seed Plants*, 2nd Edn New York, NY: Wiley.

[B38] Liu T. Ohashi-Ito K. Bergmann D. C. (2009). Orthologs of *Arabidopsis thaliana* stomatal bhlh genes and regulation of stomatal development in grasses. *Development* 136 2265–2276. 10.1242/dev.032938 19502487

[B39] MacAlister C. A. Ohashi-Ito K. Bergmann D. C. (2007). Transcription factor control of asymmetric cell divisions that establish the stomatal lineage. *Nature* 445 537–540. 10.1038/nature05491 17183265

[B40] Majore I. Wilhelm B. Marten I. (2002). Identification of K+ channels in the plasma membrane of maize subsidiary cells. *Plant Cell Physiol.* 43 844–852. 10.1093/pcp/pcf104 12198186

[B41] Metcalfe C. R. Chalke L. (1957). *Anatomy of the Dicotyledons: Leaves, Stem and Wood in Relation to Taxonomy Wiht Notes on Economic Uses*, Vol. I London: Oxford University Press.

[B42] Munemasa S. Hauser F. Park J. Waadt R. Brandt B. Schroeder J. I. (2015). Mechanisms of abscisic acid-mediated control of stomatal aperture. *Curr. Opin. Plant Biol. Cell Biol.* 28 154–162. 10.1016/j.pbi.2015.10.010 26599955 PMC4679528

[B43] Nadeau J. A. Sack F. D. (2002). Stomatal development in Arabidopsis. *Arabidopsis Book* 1 e0066. 10.1199/tab.0066 22303215 PMC3243354

[B44] Nunes T. D. G. Zhang D. Raissig M. Y. (2020). Form, development and function of grass stomata. *Plant J.* 101 780–799. 31571301 10.1111/tpj.14552

[B45] Palevitz B. A. Hepler P. K. (1974). The control of the plane of division during stomatal differentiation in allium. I. I. Spindle Reorientation. *Chromosoma* 46 297–326. 10.1007/BF00284884

[B46] Pant D. D. (1965). On the ontogeny of stomata and other homologous structures. *Plant Sci. Ser.* 1:1024.

[B47] Pant D. D. Mehra B. (1964). Development of stomata in leaves of three species of cycas and Ginkgo Biloba L. *Bot. J. Linnean Soc.* 58 491–497. 10.1111/j.1095-8339.1964.tb00917.x

[B48] Papanatsiou M. Amtmann A. Blatt M. R. (2017). Stomatal clustering in begonia associates with the kinetics of leaf gaseous exchange and influences water use efficiency. *J. Exp. Bot.* 68 2309–2315. 10.1093/jxb/erx072 28369641 PMC5447881

[B49] Pickett-Heaps J. D. (1969). Preprophase microtubules and stomatal differentiation; some effects of centrifugation on symmetrical and asymmetrical cell division. *J. Ultrastruct. Res.* 27 24–44. 10.1016/S0022-5320(69)90018-90015 5769725

[B50] Pillitteri L. J. Dong J. (2013). Stomatal development in Arabidopsis. *Am. Soc. Plant Biol.* 11:e0066. 10.1199/tab.0162 23864836 PMC3711358

[B51] Pillitteri L. J. Sloan D. B. Bogenschutz N. L. Torii K. U. (2007). Termination of asymmetric cell division and differentiation of stomata. *Nature* 445, 501–505. 10.1038/nature05467 17183267

[B52] Prabhakar M. (2004). Structure, delimitation, nomenclature and classification of stomata. *Acta Bot. Sin.* 46 242–252.

[B53] Raissig M. T. Abrash E. Bettadapur A. Vogel J. P. Bergmann D. C. (2016). Grasses use an alternatively wired BHLH transcription factor network to establish stomatal identity. *Proc. Natl. Acad. Sci. U.S.A.* 113 8326–8331. 10.1073/pnas.1606728113 27382177 PMC4961163

[B54] Raissig M. T. Matos J. L. Gil M. X. A. Kornfeld A. Bettadapur A. Abrash E. (2017). Mobile MUTE specifies subsidiary cells to build physiologically improved grass stomata. *Science* 355 1215–1218. 10.1126/science.aal3254 28302860

[B55] Raschke K. Fellows M. P. (1971). Stomatal movement in zea mays: shuttle of potassium and chloride between guard cells and subsidiary cells. *Planta* 101 296–316. 10.1007/BF00398116 24488474

[B56] Rasmussen H. (1981). Terminology and classification of stomata and stomatal development—a critical survey. *Bot. J. Linnean Soc.* 83 199–212. 10.1111/j.1095-8339.1981.tb0093.x

[B57] Roth-Nebelsick A. Hassiotou F. Veneklaas E. J. (2009). Stomatal crypts have small effects on transpiration: a numerical model analysis. *Plant Physiol.* 151 2018–2027. 10.1104/pp.109.146969 19864375 PMC2785996

[B58] Rudall J. Hilton J. Bateman M. (2013). Several developmental and morphogenetic factors govern the evolution of stomatal patterning in land plants. *New Phytol.* 200 598–614. 23909825 10.1111/nph.12406

[B59] Rudall P. J. Julier A. C. M. Kidner C. A. (2018). Ultrastructure and development of non-contiguous stomatal clusters and helicocytic patterning in begonia. *Ann. Bot.* 122 767–776. 10.1093/aob/mcx146 29186307 PMC6215052

[B60] Rui Y. Anderson C. T. (2016). Functional analysis of cellulose and xyloglucan in the walls of stomatal guard cells of Arabidopsis. *Plant Physiol.* 170 1398–1419. 10.1104/pp.15.01066 26729799 PMC4775103

[B61] Sack F. (1987). Development and structure of stomata,” in *Stomatal Function*, eds Zeiger E. Farquhar G. D. Cowan I. R. (Stanford: Stanford University Press).

[B62] Sampaio V. S. Araújo N. D. Agra M. F. (2014). Characters of leaf epidermis in Solanum (Clade Brevantherum) Species from Atlantic Forest of Northeastern Brazil. *South Afr. J. Bot.* 94 108–113. 10.1016/j.sajb.2014.06.004

[B63] Serna L. Fenoll C. (2000). Stomatal development and patterning in Arabidopsis Leaves. *Physiol. Plant.* 109 351–358. 10.1034/j.1399-3054.2000.100317.x 11841302

[B64] Shao W. Dong J. (2016). Polarity in plant asymmetric cell division: division orientation and cell fate differentiation. *Dev. Biol.* 419 121–131. 10.1016/j.ydbio.2016.07.020 27475487 PMC5522747

[B65] Sharpe P. J. H. Wu H.-I. Spence R. D. (1987). Stomatal mechanics,” in *Stomatal Function*, eds Zeiger E. Farquhar G. D. Cowan I. R. (Stanford: Stanford University Press), 91–114.

[B66] Shpak E. D. Berthiaume C. T. Hill E. J. Torii K. U. (2004). Synergistic interaction of Three ERECTA-family receptor-like kinases controls arabidopsis organ growth and flower development by promoting cell proliferation. *Development* 131 1491–1501. 10.1242/dev.01028 14985254

[B67] Shtein I. Shelef Y. Marom Z. Zelinger E. Schwartz A. Popper Z. A. (2017). Stomatal Cell wall composition: distinctive structural patterns associated with different phylogenetic groups. *Ann. Bot.* 119 1021–1033. 10.1093/aob/mcw275 28158449 PMC5604698

[B68] Simmons A. R. Bergmann D. C. (2016). Transcriptional control of cell fate in the stomatal lineage. *Curr. Opin. Plant Biol. Growth Dev.* 29 1–8. 10.1016/j.pbi.2015.09.008 26550955 PMC4753106

[B69] Stace C. A. (1965). Cuticular studies as an aid to plant taxonomy. *Bull. Br. Museum* 4 37–40.

[B70] Stebbins G. L. Shah S. S. (1960). developmental studies of cell differentiation in the epidermis of monocotyledons: II. Cytological Features of Stomatal Development in the Gramineae. *Dev. Biol.* 2 477–500.

[B71] Tomlinson P. B. (1969). *Anatomy of the Monocotyldeons. III Commelinales - Zingiberales.* Oxford: Clarendon Press.

[B72] Tomlinson P. B. (1974). Development of the stomatal complex as a taxonomic character in the monocotyledons. *TAXON* 23 109–128. 10.2307/1218094

[B73] Torii K. U. (2015). Stomatal differentiation: the beginning and the end. *Curr. Opin. Plant Biol. Cell Biol.* 28 16–22. 10.1016/j.pbi.2015.08.005 26344486

[B74] Vico G. Manzoni S. Palmroth S. Katul G. (2011). Effects of stomatal delays on the economics of leaf gas exchange under intermittent light regimes. *New Phytol.* 192 640–652. 10.1111/j.1469-8137.2011.03847.x 21851359

[B75] Wang H. Guo S. Qiao X. Guo J. Li Z. Zhou Y. (2019a). BZU2/ZmMUTE Controls symmetrical division of guard mother cell and specifies neighbor cell fate in maize. *PLoS Genet.* 15:e1008377. 10.1371/journal.pgen.1008377 31465456 PMC6738654

[B76] Wang H. Yan S. Xin H. Huang W. Zhang H. Teng S. (2019b). A subsidiary cell-localized glucose transporter promotes stomatal conductance and photosynthesis. *Plant Cell* 31 1328–1343. 10.1105/tpc.18.00736 30996077 PMC6588317

[B77] Willmer C. M. Pallas J. E. Jr. (1972). A survey of stomatal movements and associated potassium fluxes in the plant kingdom. *Can. J. Bot.* 51 37–24.

[B78] Xu M. Chen F. Qi S. Zhang L. Wu S. (2018). Loss or duplication of key regulatory genes coincides with environmental adaptation of the stomatal complex in Nymphaea Colorata and Kalanchoe Laxiflora. *Horticult. Res.* 5 1–16. 10.1038/s41438-018-0048-48 PMC6068134 30083357

[B79] Yi H. Rui Y. Kandemir B. Wang J. Z. Anderson C. T. Puri V. M. (2018). Mechanical effects of cellulose, xyloglucan, and pectins on stomatal guard cells of Arabidopsis Thaliana. *Front. Plant Sci.* 9:1566. 10.3389/fpls.2018.01566 30455709 PMC6230562

[B80] Zeiger E. Stebbins G. L. (1972). Developmental genetics in barley: a mutant for stomatal development. *Am. J. Bot.* 59 143–148.

[B81] Zhang X. Facette M. Humphries J. A. Shen Z. Park Y. Sutimantanapi D. (2012). Identification of PAN2 by quantitative proteomics as a leucine-rich repeat–receptor-like kinase acting upstream of PAN1 to polarize cell division in maize. *Plant Cell* 24 4577–4589. 23175742 10.1105/tpc.112.104125 PMC3531853

[B82] Ziegler H. (1987). The evolution of stomata,” in *Stomatal Function*, eds Zeiger E. Farquhar G. D. Cowan I. R. (Stanford: Stanford University Press), 29–58.

